# Hidden Intruder: A Rare Encounter of Dirofilaria Masquerading as a Breast Lump

**DOI:** 10.7759/cureus.50535

**Published:** 2023-12-14

**Authors:** Sumin V Sulaiman, Ravindran Chirukandath, Sharath K Krishnan, Rajesh M Subramanian, Shahina Salim Aysha

**Affiliations:** 1 General Surgery, Government Medical College, Thrissur, IND; 2 Surgical Oncology, Government Medical College, Thrissur, IND; 3 Surgery, Government Medical College, Thrissur, IND

**Keywords:** dirofilaria, parasitic, birads, mammogram, breast lump

## Abstract

*Dirofilaria*, commonly known as heartworm, is a parasitic nematode that primarily infects canines. However, human infections have been reported and can present as subcutaneous nodules in different parts of the body. We present a case of a 43-year-old female who presented with a breast lump that was ultimately diagnosed as a *Dirofilaria* infection, a rare occurrence in humans. This case report shows that considering parasites in unusual presentations is of utmost importance, especially in regions known to have a high prevalence of such infections.

## Introduction

*Dirofilaria immitis* is a mosquito-borne parasitic nematode that primarily infects canines, causing heartworm disease. Although human infections are rare, they have been reported in regions endemic to *Dirofilaria*. Human dirofilariasis is caused mainly by *Dirofilaria* belonging to two species: *Dirofilaria repens* and *Dirofilaria immitis* [1], and it usually presents as subcutaneous nodules and pulmonary lesions [2]. *Dirofilaria* presenting as a breast lump is rare and occurs as solid or mostly cystic lesions [3]. Given the potential for misdiagnosis, clinicians need to consider parasitic infections when evaluating patients with unusual breast lumps.

## Case presentation

**Figure 1:**
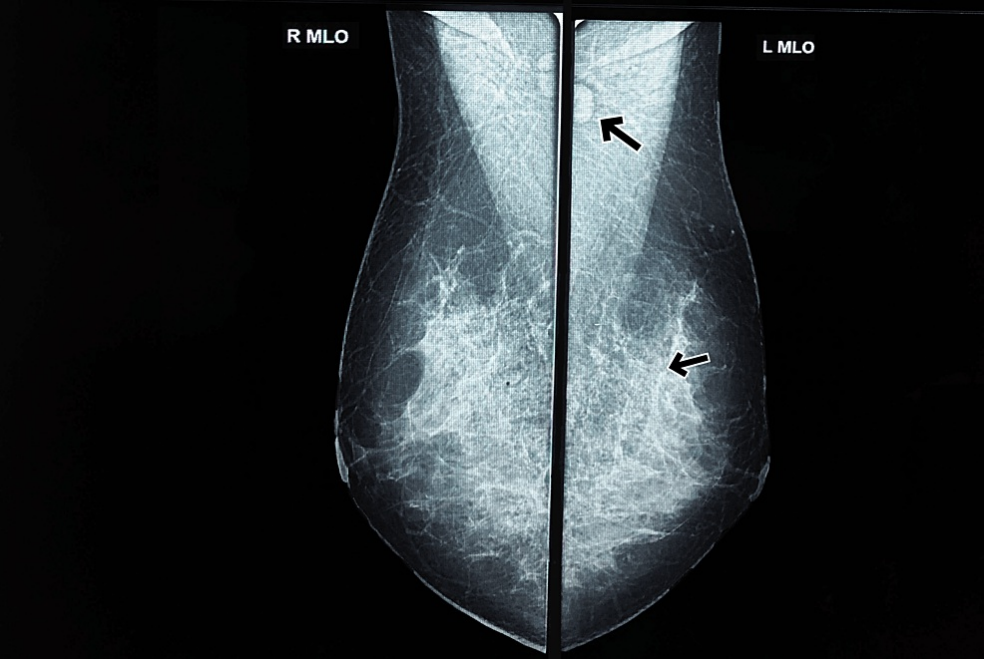
Mammogram Dense breast tissue with benign axillary lymph node

A 43-year-old female from mid-Kerala, South India, presented to the clinic with a painless lump in her left breast that had been present for three weeks. She had no significant medical history. She is of low socioeconomic status, and there are many mosquito breeding places in and around her house, and there are dogs in her neighbor's home. Her mother died of carcinoma lung, and her mother's sister was diagnosed with carcinoma breast. Physical examination revealed a firm, mobile, non-tender lump measuring 2 x 1 cm located in the upper inner quadrant of the left breast with a benign-looking lymph node in the left axilla. Considering the patient's age, family history, and the presence of a palpable breast mass, a mammogram and fine-needle aspiration cytology (FNAC) were performed. The mammogram (Figure 1) with ultrasonogram correlation showed a well-defined hypoechoic lesion with multiple internal septations and echogenic areas - breast imaging-reporting and data system (BI-RADS 4A).

**Figure 2:**
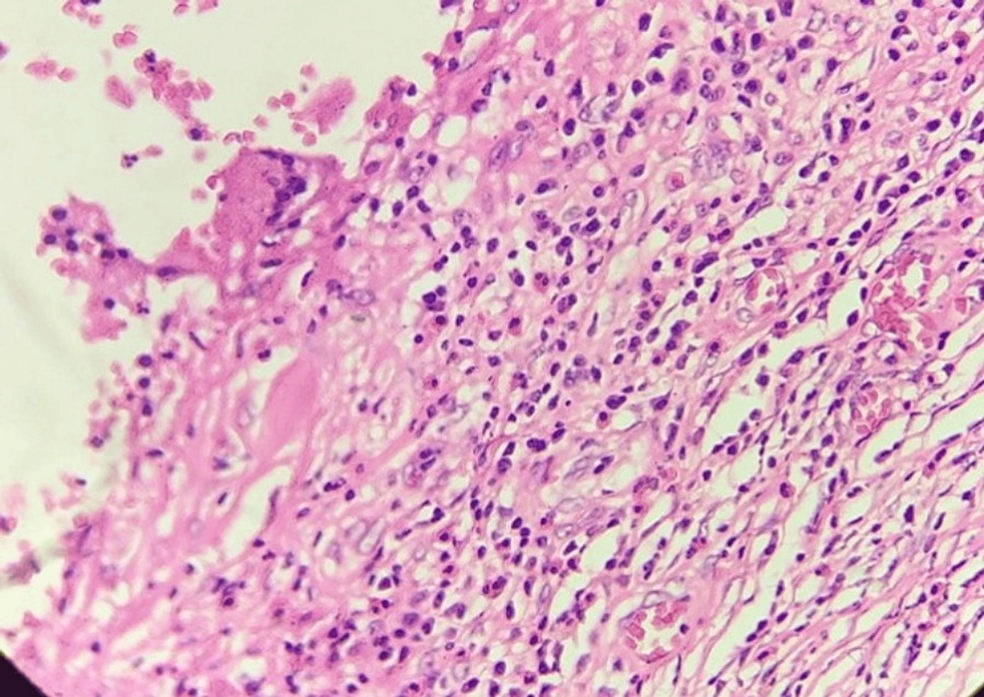
Histopathology H&E stain with 400x magnification showing numerous eosinophils and plasma cells in the surrounding fibrous tissue close to the parasite

**Figure 3:**
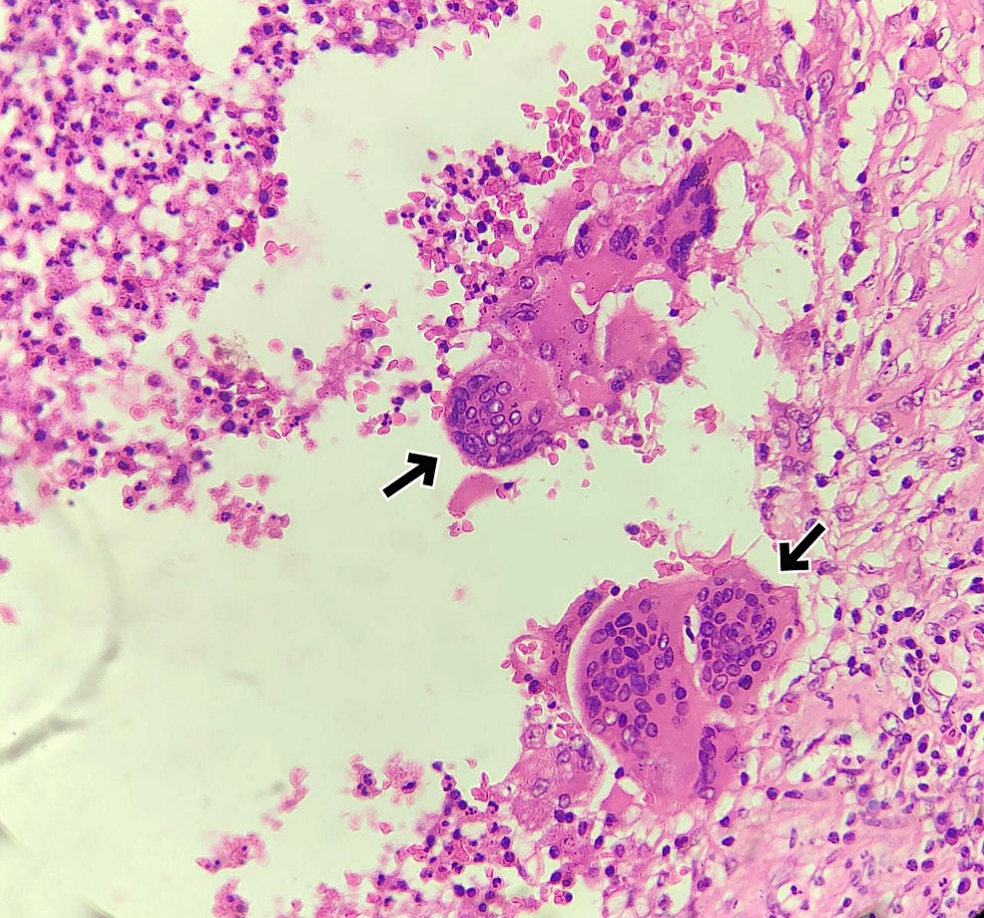
Histopathology 100x magnification, H &E stain showing multinucleated foreign body giant cells close to the parasite

**Figure 4:**
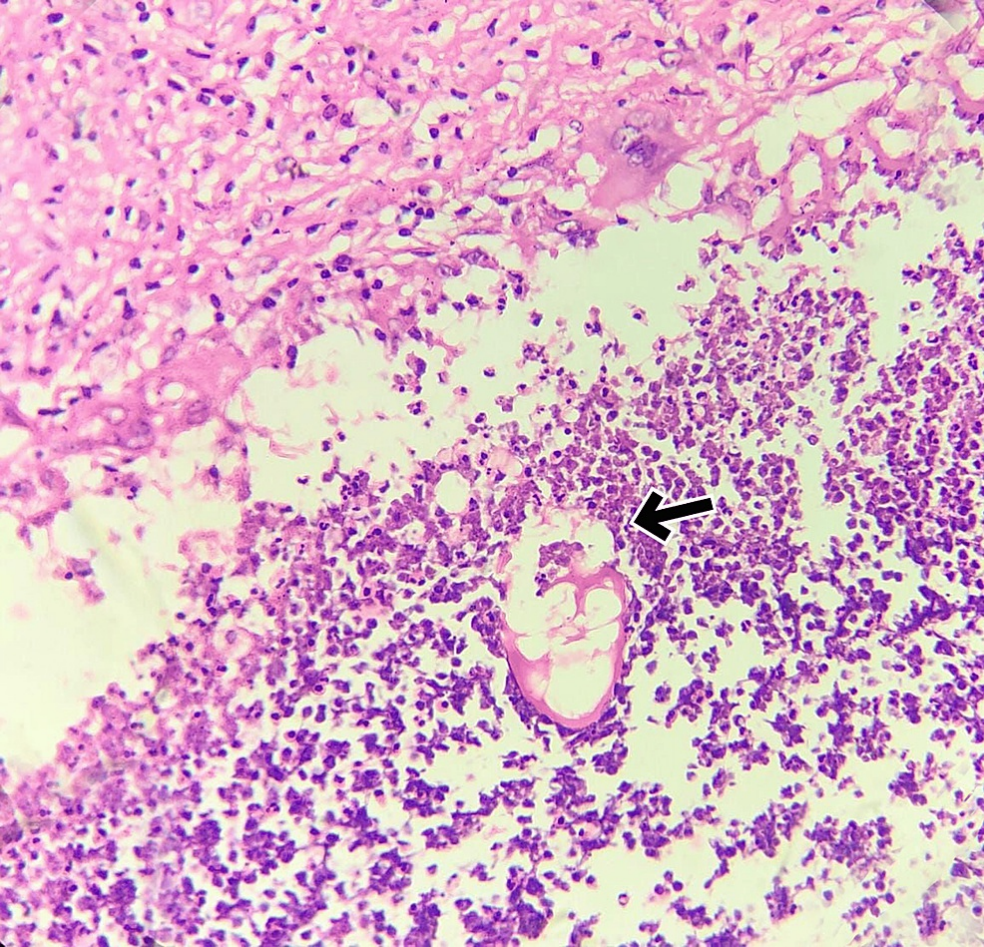
Microscopy 400x magnification, H&E stain showing a cross-section of the worm

FNAC revealed a suppurative inflammatory lesion, and the sample revealed no malignant cells, indicating a benign nature. Her blood count was normal. Given the patient's persistent concern and the atypical appearance of the mass, a wide local excision was performed. Histopathological examination revealed greyish-white firm soft tissue measuring 2 x 2 cm and oval in shape. The cut section surprisingly showed a thread-like worm 1 cm long. On microscopy, the lesion demonstrated a chronic inflammatory lesion (Figure 2) with suppuration and a foreign body giant cell reaction (Figure 3). A cross-section of the worm is seen (Figure 4), morphologically consistent with *Dirofilaria*. Subsequent evaluation of the patient's serology confirmed the diagnosis of *Dirofilaria immitis* infection. No complications or recurrences were observed during the follow-up period.

## Discussion

Dirofilariasis is a zoonotic infection transmitted to humans through mosquito bites. Human dirofilariasis is an infrequent helminthic infection caused by filarial worms of the *Dirofilaria* species, with occasional zoonotic transmission. These worms naturally parasitize dogs, cats, foxes, and various wild mammals [4]. Transmission to humans occurs through mosquitoes. While most cases of dirofilariasis are asymptomatic, symptomatic individuals typically present with subcutaneous nodules or lung parenchymal disease.

In India, where most documented cases of human dirofilariasis occur, ocular infections are prevalent [5,6]. *Dirofilaria repens* is the primary causative agent in subcutaneous human dirofilariasis in India and the Asian subcontinent. Coastal Kerala is particularly endemic to dirofilariasis due to the presence of suitable vectors and climatic conditions [4]. While *Dirofilaria repens* is the predominant cause in the northern and western parts of India [7], there have been a few reported cases of *Dirofilaria immitis* [8]. The mosquitoes *Culex*, *Aedes*, and even *Anopheles* species form the vector for this nematode [9].

Clinically, human dirofilariasis poses challenges as subcutaneous lesions may be initially misidentified as malignant tumors. This misidentification often leads to invasive investigations and surgeries before an accurate diagnosis is established. The pathology of the condition arises from the abnormal localization of immature worms intended for nonhuman hosts.

The incidence of zoonotic filariasis is increasing worldwide, possibly attributed to global warming and climate change [1,10-12]. Studies have demonstrated that elevated temperatures can accelerate larval stage 3 development in mosquitoes, shorten their developmental period, modify seasonal transmission patterns, influence mosquito feeding behavior, and expand breeding areas [1,13]. Although the lungs and subcutaneous tissues are the most common sites of involvement [14], breast involvement is extremely rare. The clinical presentation of dirofilariasis can mimic various subcutaneous conditions, including breast masses, leading to potential misdiagnosis. The subcutaneous nodules, which may be located anywhere in the body, often evoke suspicion of tumor growth, necessitating an excisional biopsy to exclude malignancy [15,16].

In this case, the patient presented with a breast lump, which was initially considered to be a benign fibroepithelial lesion based on FNAC. However, further investigations revealed the presence of *Dirofilaria immitis*, highlighting the importance of considering parasitic infections in the differential diagnosis of breast masses, particularly in regions where the parasite is endemic.

Histopathological examination remains crucial in establishing the diagnosis of dirofilariasis. However, serological testing can help confirm the infection. Treatment options for human dirofilariasis include surgical excision, antiparasitic medications, or a combination of both.

## Conclusions

This case report emphasizes the importance of considering parasitic infections, such as *Dirofilaria immitis*, in the differential diagnosis of breast masses. Clinicians should maintain a high index of suspicion, especially in areas where the parasite is endemic. Histopathological examination and serological testing are essential for confirming the diagnosis. Prompt diagnosis and appropriate management can lead to favorable outcomes for patients presenting with this rare manifestation of dirofilariasis.
